# Radiation-Induced Asymmetric Grafting of Different Monomers into Base Films to Prepare Novel Bipolar Membranes

**DOI:** 10.3390/molecules26072028

**Published:** 2021-04-02

**Authors:** Shin-ichi Sawada, Yasunari Maekawa

**Affiliations:** Takasaki Advanced Radiation Research Institute, Quantum Beam Science Directorate, National Institutes for Quantum and Radiological Science and Technology (QST), 1233 Watanuki-machi, Takasaki-Shi 370-1292, Japan; maekawa.yasunari@qst.go.jp

**Keywords:** bipolar membrane, radiation induced asymmetric grafting, phase separation

## Abstract

We prepared novel bipolar membranes (BPMs) consisting of cation and anion exchange layers (CEL and AEL) using radiation-induced asymmetric graft polymerization (RIAGP). In this technique, graft polymers containing cation and anion exchange groups were introduced into a base film from each side. To create a clear CEL/AEL boundary, grafting reactions were performed from each surface side using two graft monomer solutions, which are immiscible in each other. Sodium *p*-styrenesulfonate (SSS) and acrylic acid (AA) in water were co-grafted from one side of the base ethylene-co-tetrafluoroethylene film, and chloromethyl styrene (CMS) in xylene was simultaneously grafted from the other side, and then the CMS units were quaternized to afford a BPM. The distinct SSS + AA- and CMS-grafted layers were formed owing to the immiscibility of hydrophilic SSS + AA and hydrophobic CMS monomer solutions. This is the first BPM with a clear CEL/AEL boundary prepared by RIAGP. However, in this BPM, the CEL was considerably thinner than the AEL, which may be a problem in practical applications. Then, by using different starting times of the first SSS+AA and second CMS grafting reactions, the CEL and AEL thicknesses was found to be controlled in RIAGP.

## 1. Introduction

Membrane separation technologies have been applied in various processes such as seawater desalination, water purification, liquid product condensation, gas molecule collection, and electric power generation, due to the compact module system, simplicity for operation, and low energy consumption [1,2,3]. The most widely-used application is pressure-driven water filtration using porous membranes, which allow water to pass through but not impurities by the size exclusion effect [4,5]. They are categorized into microfiltration, nanofiltration, ultrafiltration, and reverse osmosis, in descending order of the membrane pore size. 

Ion exchange membranes (IEMs) are one of the representative non-porous functional membranes and roughly classified into cation exchange membranes (CEMs) and anion exchange membranes (AEMs) [6]. In the hydration state, counter ions (cations for a CEM and anions for an AEM) can freely move throughout the membrane, while co-ions (anions for a CEM and cations for an AEM) are excluded from the membrane by the repulsion interaction with immobilized ionic groups. CEMs and AEMs are used in the chlor-alkali process [7], seawater condensation for table salt production [8], and polymer electrolyte fuel cells [9,10]. 

A bipolar membrane (BPM) is a special kind of IEM and is composed of a cation exchange layer (CEL) and anion exchange layer (AEL), which work as a CEM and AEM, respectively [11,12,13,14,15,16,17]. The characteristic phenomena occurring inside a BPM are water dissociation (H_2_O → H^+^ + OH^−^) and water formation (H^+^ + OH^−^ → H_2_O) at the CEL/AEL boundary. The water dissociation property is significantly useful for practical use because it enables simultaneous generation of acid and base by electrodialysis. Specifically, for example, the following BPM electrodialysis processes were researched and developed: recovery of acetic acid from acetaldehyde wastewater [11], regeneration of HBr and NaOH from NaBr wastewater [12], production of HCl and NaOH from a NaCl solution [13], and generation of HCl and NH_3_ from a NH_4_Cl solution [14]. 

For polymer electrolyte fuel cells, one of the following two membranes is generally used: H^+^-conductive CEM or OH^−^-conductive AEM. In these cases, the membranes should be kept in a wet state for maintaining the ion conductivity, which determines the output power. For this purpose, fuel cell equipment is required to have a humidifying function. In this regard, a fuel cell employing a BPM has attracted considerable attention because of its water formation property. During the operation of this type of fuel cell, water is continuously generated at the CEL/AEL boundary to maintain a hydration state that is suitable for ion transport. This so-called self-humidification eliminates the demand for an external humidifier, which reduces the size and production cost of a fuel cell system [15,16,17]. 

A typical way to prepare a BPM is to coat anion exchange polymers on the base CEM or vice versa (i.e., a combination of cation exchange coating polymers and a base AEM) using the solution-casting method [18]. However, this conventional BPM has a potential issue that the adhesiveness between the CEL and AEL is gradually weaken during long-term operation, which deteriorates electrochemical performance. Therefore, novel BPMs possessing high mechanical strength are urgently needed. 

A promising technique for preparing functional polymer membranes is a radiation-induced grafting method [19,20]. In this method, a base polymer film is irradiated by a γ-ray or electron beam, and then the film is immersed in a monomer solution. Graft polymerization of monomers is initiated at the polymer backbones of the film from both sides of surfaces and gradually propagates deep inside to extend the grafted regions, which finally results in the homogeneous distribution of graft chains throughout the film. Then, if needed, graft chains are chemically transformed to functional polymers to obtain the required membranes. To prepare CEMs, three routes are mainly used: (i) grafting of sodium *p*-styrenesulfonate (SSS), (ii) grafting of ethyl *p*-styrenesulfonate (EtSS) and subsequent hydrolysis of EtSS units, and (iii) grafting of styrene and subsequent sulfonation of styrene units. Regarding (i), acrylic acid (AA) is sometimes co-grafted because it accelerates the SSS grafting [21]. For the preparation of AEMs, the most well-known way is the grafting of chloromethylstyrene (CMS) and subsequent quaternization of CMS units. 

In this study, we propose radiation-induced “asymmetric” grafting polymerization (RIAGP) for different monomers to produce monolithic BPMs. Specifically, the monomer for CEL formation (e.g., SSS and EtSS) is radiation-grafted from one side of the base film, and the monomer for AEL formation (e.g., CMS) is grafted from the other side. If the CEL and AEL are phase-separated from each other with a clear boundary, the prepared membrane works as a BPM. The RIAGP technique is expected to overcome the aforementioned disadvantage of conventional BPMs. Namely, this novel monolithic BPM is hard to be decomposed because all the graft chains that comprise the CEL and AEL are covalently bonded to the main chains of the same base film. 

To date, some researchers have prepared BPMs based on porous films by RIAGP, but they did not report their detailed structures [22,23,24]. Guan et al. have recently used non-porous base films to prepare BPMs and used them for fuel cell tests [25]. The fuel cell was demonstrated to work even under dry conditions owing to the self-humidification ability, although the output power was not high. In this BPM, unfortunately, the CEL and AEL were not clearly separated from each other according to an energy dissipative X-ray (EDX) analysis. If the boundary between the CEL and AEL is clear, water formation at the boundary will efficiently occur to improve the output power. 

The objective of this study is to create monolithic BPMs with a clear CEL/AEL boundary by RIAGP. First, as a preliminary experiment, the solubility of various graft monomers in solvents and the reactivity of monomers in graft polymerization into base films were examined. This provided us with information on the proper combination of base films, monomers, and solvents. Next, RIAGP was performed to obtain two-layered grafted films, and their structure was investigated using a scanning electron microscope (SEM) coupled with EDX. Finally, BPMs were characterized in terms of cation exchange capacity (*CEC*), anion exchange capacity (*AEC*), and water uptake. 

## 2. Experimental

### 2.1. Materials and Chemicals

A poly(vinylidene fluoride) (PVDF) film (50 μm, Kureha Chemical Co. Ltd., Tokyo, Japan), poly(ethylene-*co*-tetrafluoroethylene) (ETFE) film (50 μm, AGC Co. Ltd., Tokyo, Japan), polyethylene (PE) film (38 μm, Saxin Co. Ltd., Otsu, Japan), and Nylon 6 film (25 μm, Unitika Co. Ltd., Osaka, Japan) were used as a base material for radiation grafting. SSS and AA were purchased from FUJIFILM Wako Pure Chemical Co. Ltd. (Osaka, Japan). EtSS was purchased from Tosoh Finechem Co. Ltd. (Tokyo, Japan). CMS was purchased from AGC Seimi Chemical Co. Ltd. (Chigasaki, Japan) and purified by the extraction of a polymerization inhibitor in a 1 mol/L aqueous NaOH solution before use. Trimethylamine (TMA), methanol, ethanol, 1-pentanol, DMSO, N-methylpyrrolidone (NMP), toluene, and xylene were purchased from FUJIFILM Wako Pure Chemical Co. Ltd. (Osaka, Japan). Deionized water was produced by the Milli-Q water purification system (Merck Millipore Co. Ltd., Burlington, MA, USA). 

### 2.2. Radiation-Induced Grafting of Several Monomers into Base Films

A base film with a size of 2 × 2 cm^2^ was wiped with acetone to remove any impurities on the film surface and placed in a glass ampoule with a stopcock. The ampoule was evacuated for 1 h to remove air trapped inside the film and then filled with Ar gas, followed by closing the stopcock. The film enclosed in the ampoule was irradiated with 80-kGy ^60^Co γ-rays (Cell No. 6, National Institutes for Quantum and Radiological Science and Technology (QST) at Takasaki, Japan) at room temperature. After irradiation, the ampoule was vacuumed for 10 min to remove decomposition products. 

The irradiated film was exposed to air for 1 min and placed in the glass ampoule again. Ar gas was introduced into the ampoule via a thin needle to prevent air entry while the stopcock was open. A monomer solution was injected into the ampoule using a syringe such that the film was fully immersed in the solution. Ar gas was bubbled into the monomer solution for deaeration for 5 min. For the RIGP reaction, the closed ampoule was placed in an oven (DKN 401, Yamato Scientific Co. Ltd., Tokyo, Japan) at 60 °C for 8 h. 

After the RIGP reaction, the film was removed from the ampoule and immersed in a proper solvent at 60 °C for approximately 6 h to remove any remaining monomers and homopolymers. The film was dried in a vacuum oven at 50 °C for 12 h. Then, the degree of grafting (*DOG*) was calculated using:

(1) $$DOG\;=\;\left(W_{1}-\;W_{0}\right)/W_{0}$$

where *W*_0_ and *W*_1_ are the weights of the original and grafted films, respectively. 

### 2.3. RIAGP of Monomers into Base Films

**Figure 1:**
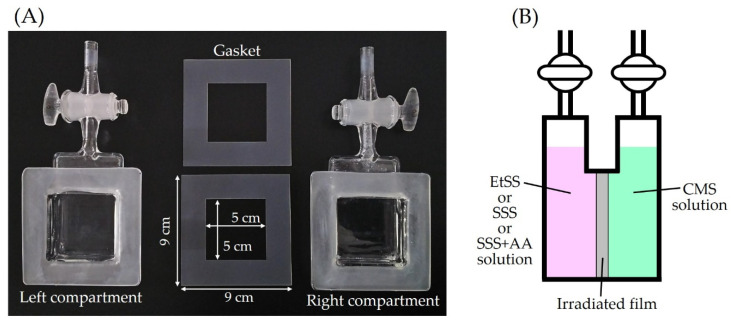
(**A**): Picture of the two-compartment glass cell for radiation-induced asymmetric graft polymerization (RIAGP). The irradiated film was located between two gaskets. (**B**): Schematic diagram of RIAGP. Two different monomer solutions were injected into the left and right compartments.

The 80-kGy γ-ray (5 kGy/h) irradiation of the base film with a size of 9 × 9 cm^2^ was performed according to the procedure described in Section 2.2. After the irradiation, the ampoule was evacuated for 15 min to remove decomposition products. The film was removed from the ampoule and positioned at the center of a homemade two-compartment glass cell with two stopcocks shown in Figure 1A. The effective graft reaction area, at which the film was in contact with monomer solutions, had a size of 25 cm^2^ (= 5 × 5 cm^2^). Ar gas was introduced into the left and right compartments at the same time to prevent air entry. As shown in Figure 1B, a solution of monomers (EtSS, SSS, or a mixture of SSS + AA) was injected into the left compartment, and a CMS monomer solution was similarly injected into the right one. After Ar bubbling for 10 min, the stopcocks of both compartments were closed. The reaction cell was placed in an oven (DKN 401, Yamato Scientific Co. Ltd., Tokyo, Japan) at 60 °C for RIAGP for 6 h. 

After the RIAGP reaction, the film was removed from the cell and immersed in a proper solvent at 60 °C for approximately 6 h to remove any remaining monomers and homopolymers. Then, the film was dried in a vacuum oven at 40 °C for 12 h. The *DOG* was calculated using Equation (1). 

### 2.4. Characterization of Grafted Films and BPMs

The grafted film was cut into 5 × 6 mm^2^ pieces and immersed in epoxy resin that was prepared by mixing 2.3 mL of Epok 812, 1.5 mL of dodecenyl succinic anhydride (DDSA), 1.2 mL of methyl nadic anhydride (MNA), and 75 μL of tridimethyl aminomethyl phenol (DMP-30) (Okenshoji Co. Ltd., Tokyo, Japan). The film sample embedded in epoxy resin was heated at 80 °C for 24 h for solidification. The solidified resin was sliced little by little using a slicing machine (RM 2145, Leica Microsystems Co. Ltd., Wetzlar, Germany) to expose the cross-sectional surface of the film. The prepared sample was sputter-coated with Pt to improve image resolution and to avoid electrical charging. The film morphology was observed by SEM (JSM-5600, JEOL Co. Ltd., Tokyo, Japan), and the distribution of sulfur and chlorine in the transverse plane of the film was analyzed using an EDX spectrometer (X-Max N50, HORIBA Co. Ltd., Kyoto, Japan). The structure of the grafted film was studied by attenuated total reflectance (ATR) Fourier transform infrared (FT-IR) spectroscopy. Spectral data were recorded using an FT-IR spectrometer (FT-710, HORIBA Co. Ltd., Kyoto, Japan) in the range of 600–1800 cm^−1^ with a resolution of 4 cm^−1^. 

The grafted films were immersed in a 30% TMA aqueous solution for 8 h for the quaternization of the CMS units. The obtained BPM was immersed in deionized water for 24 h. The cross-sectional morphology of the BPM was observed by SEM, and the distribution of sulfur and chlorine atoms was analyzed by EDX, as described above. The water uptake measurement for the BPM was performed when the counter cations and anions were H^+^ and Cl^−^, respectively (H^+^/Cl^−^ form). The BPM was immersed in deionized water at 25 °C for 24 h, and its weight, *W_W_*, was measured. Water uptake was calculated using:

(2) $$\mathrm{Water}\;\mathrm{uptake}\;=\;100\times \left(W_{W}-\;W_{D}\right)/W_{D}$$

where *W_D_* is the weight of the H^+^/Cl^−^ form BPM in a vacuum-dried state. The technique for measuring the *CEC* and *AEC* of the BPM is described in Section 3.3. 

## 3. Results and Discussion

### 3.1. Graft Polymerization for the CEL and AEL

#### 3.1.1. Preparation Routes for BPMs

**Figure 2:**
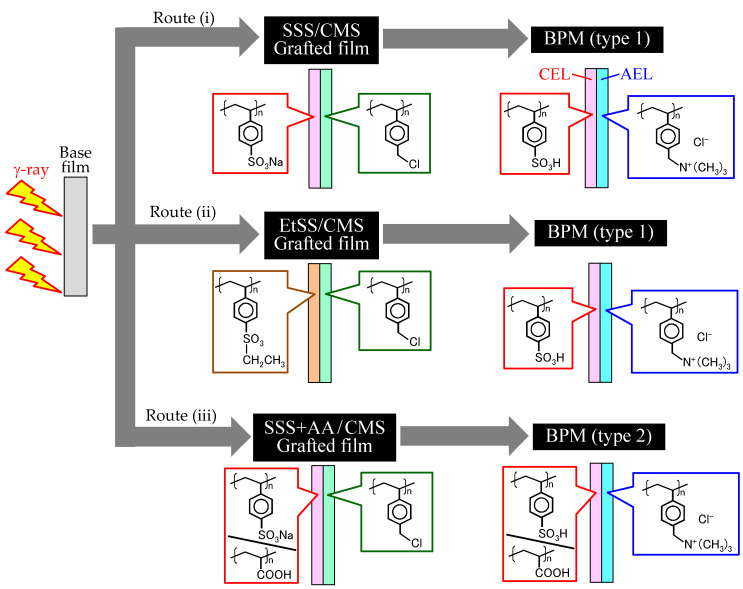
Three potential routes (i)–(iii) for the preparation of bipolar membranes (BPMs) by RIAGP.

Figure 2 shows three potential routes for BPM preparation by RIAGP. In Routes (i)–(iii), SSS, EtSS, and SSS + AA are grafted from the left side of the base film. In Route (ii), the EtSS units are hydrolyzed to form the CEL. In all routes, CMS is grafted from the right side of the base film, and CMS units are quaternized to form the AEL. Routes (i) and (ii) produce a type-1 BPM, whereas Route (iii) produces a type-2 BPM. The difference between them is that the latter BPM contains not only poly(styrene sulfonic acid) graft chains but also poly(acrylic acid) graft chains. 

In a previous study, a BPM was prepared using the following steps: radiation grafting of styrene and 1-vinylimidazole (VIm) from each side of the base film, sulfonation of styrene units to form the CEL, and alkylation of VIm units to form the AEL [25]. However, this procedure has two issues: the possibility of film degradation by a strong acid agent used for sulfonation (pointed out in Ref [25]) and the relatively low basicity of the introduced imidazolium groups. These issues can be avoided in Routes (i)–(iii), as shown in Figure 2, because a strong acid agent is not used, and quaternary ammonium groups have sufficiently high basicity. 

#### 3.1.2. Solubility of Monomers in Solvents 

For the clear phase separation of two grafted regions propagating from both sides of the base film, the chemical properties of two grafting monomer solutions, i.e., hydrophilic/hydrophobic nature, should be controlled by a solvent. A standard measure for the hydrophilic and hydrophobic properties is the octanol/water partition coefficient (P_OW_) that is evaluated by a simple experiment. P_OW_ is defined as the ratio of the solvent’s concentration in octanol and water phases, which represents the hydrophilicity (or hydrophobicity) of a solvent. Table 1 shows the values of Log(P_OW_) of commonly used solvents [26]. The Log(P_OW_) values of DMSO and water are very low owing to their hydrophilicity. By contrast, those of toluene and xylene are very high owing to their hydrophobicity. Accordingly, DMSO and water were used to prepare a hydrophilic monomer solution, and xylene was used to prepare a hydrophobic monomer solution. 

The solubility of monomers in solvents was examined for the preparation of monomer solutions for grafting. The monomer was added to a solvent at a certain concentration, and this mixture was stirred. Visual inspection was used to check whether the monomer was completely miscible or not. Table 2 shows the results of the monomer/solvent mixture tests. SSS was dissolved in water and DMSO with a concentration below 0.8 and 1.0 mol/L, respectively, i.e., DMSO was a better solvent for SSS than water. However, when 0.8 mol/L of AA (but not at 0.6 mol/L) was added, 1.0 mol/L of SSS was dissolved in water. EtSS and CMS were dissolved in both DMSO and xylene but not in water. 

#### 3.1.3. Radiation Grafting of Monomers into Base Polymer Films 

To examine the radiation grafting behavior of monomers, four base films (i.e., PVDF, ETFE, PE, and Nylon 6) were irradiated by γ-rays and immersed in various monomer solutions for grafting. Table 3 shows the experimental conditions and obtained *DOG*. For SSS, *DOG* strongly depended on the base films and solvents. SSS was not grafted to PVDF, ETFE, and PE but easily grafted to Nylon 6 in water. This occurred possibly owing to the hydrophilic nature of Nylon 6 compared to that of the other three films, which resulted in a smooth penetration of an SSS/water solution into the film. SSS was grafted to PVDF in DMSO but not to ETFE and PE, and SSS + AA was grafted to PVDF, ETFE, and PE in water. EtSS was grafted to all films in DMSO, and grafted to only ETFE and PE in xylene. Regardless of solvents, CMS was grafted to PVDF, ETFE, and PE but not to Nylon 6. This means that the AEL cannot be produced in Nylon 6, i.e., it is not a suitable base film for BPM preparation. The monomer grafting behaviors in ETFE and PE were similar to each other. Thus, PE was not adopted as a base film because the chemical stability of PE was lower than that of ETFE. Consequently, PVDF and ETFE were selected as base films for RIAGP performed in Section 3.2. 

### 3.2. Preparation of the Asymmetric Grafted Films by RIAGP

#### 3.2.1. RIAGP without Time Lag

**Figure 3:**
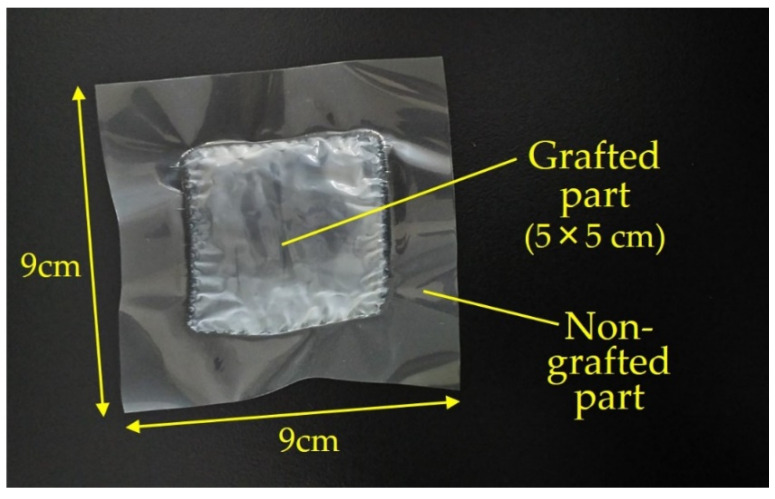
Picture of the grafted film prepared by RIAGP of sodium *p*-styrenesulfonate (SSS) and chloromethyl styrene (CMS) into a base poly(vinylidene fluoride) (PVDF) film (Run 1 in Table 4).

**Figure 4:**
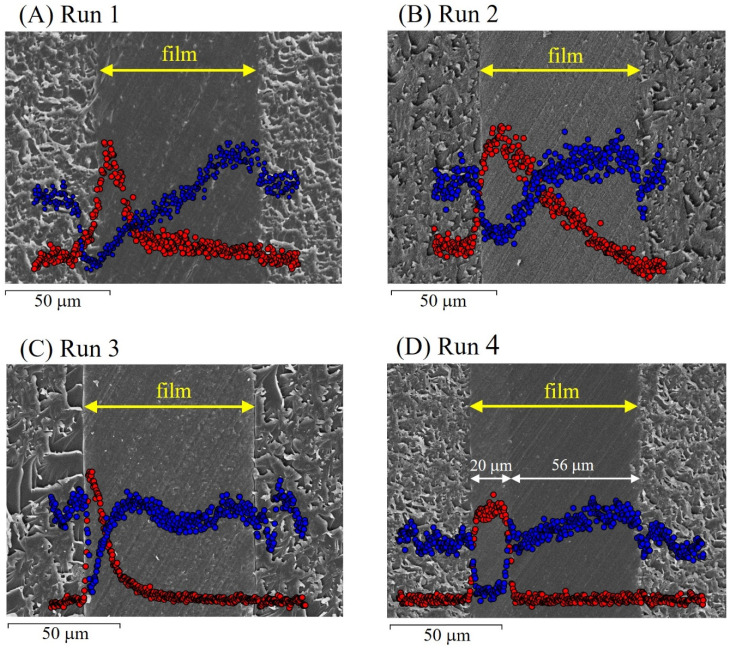
SEM images and concentration profile of sulfur (red circle) and chlorine (blue circle) of the grafted PVDF ((**A**,**B**)) and poly(ethylene-*co*-tetrafluoroethylene) (ETFE) ((**C**,**D**)) films prepared in Runs 1–4 in Table 4. Sulfur is contained in SSS and ethyl *p*-styrenesulfonate (EtSS) units, and chlorine is contained in CMS units.

The RIAGP conditions for BPM preparation were determined as listed in Table 4. In Run 1, SSS/DMSO and CMS/xylene were used as graft monomer solutions for the PVDF film (Route (i) in Figure 2). The graft polymerization of two monomers started at the same time, and this experimental condition is referred to as “no time lag”. Figure 3 shows the picture of the film after RIAGP. The central white part is the grafted part, whereas the circumference transparent part is not grafted because it was tightly sandwiched by two gaskets and monomers could not penetrate into it. The effective degree of grafting in the central grafted part, *DOG_eff_*, was calculated using:

(3) $$DOG_{eff}=\;S_{A}\times DOG/S_{B}$$

where *S_A_* and *S_B_* are the total area of the base film (81 cm^2^) and grafted area (25 cm^2^), respectively. The *DOG_eff_* of SSS and CMS into PVDF was calculated to be 26.4% from Equation (3), and the thickness of the grafted film was 56 μm. Figure 4A shows the SEM image of the grafted film and the concentration profile of sulfur and chlorine measured by EDX. As shown in Figure 1B, SSS and CMS were grafted from the left and right sides of the base film. Sulfur contained in SSS units existed only in the region near the left film surface. The concentration of chlorine contained in CMS units decreased from the right to the left in the film. Unfortunately, there is no clear boundary between SSS- and CMS-grafted regions, which can be explained by taking into account the monomer dissolution behavior. In the RIAGP process, the CMS/xylene solution penetrated from the right side and reached the front of the SSS/DMSO solution in the film. Then, CMS was dissolved in DMSO, and CMS graft polymerization proceeded to the left direction. 

In Runs 2 and 3, the monomer/solvent combinations of EtSS/DMSO and CMS/xylene were chosen for PVDF and ETFE (Route (ii) in Figure 2). The grafted films prepared in Runs 2 and 3 had thicknesses of 71 and 72 μm with *DOG_eff_* of 121 and 95.8% (see Table 4), respectively. Figure 4B,C show the SEM images of the film cross-section and the concentration profile of sulfur and chlorine for EtSS and CMS units, respectively. Similarly to Figure 4A, no clear boundary of EtSS- and CMS-grafted regions was observed. Both grafted regions were overlapped because EtSS and CMS can be dissolved in both xylene and DMSO, as shown in Table 2.

In Run 4, the monomer/solvent combinations for the formation of the CEL and AEL are SSS + AA/water and CMS/xylene, respectively (Route (iii) in Figure 2). The prepared ETFE-based grafted film had a thickness of 73 μm and *DOG_eff_* of 125%. Figure 4D shows the SEM image of the film cross-section and the concentration profile of sulfur and chlorine atoms for SSS and CMS units, respectively. Sulfur was detected only in the thin left region, and chlorine was detected only in the wide right region in the film. This means that the SSS + AA- and CMS-grafted regions were clearly phase-separated from each other in the base ETFE film. It was demonstrated that a distinct two-layered structure could be created using the hydrophilic and hydrophobic monomer solutions in RIAGP, which are immiscible in each other. 

**Figure 5:**
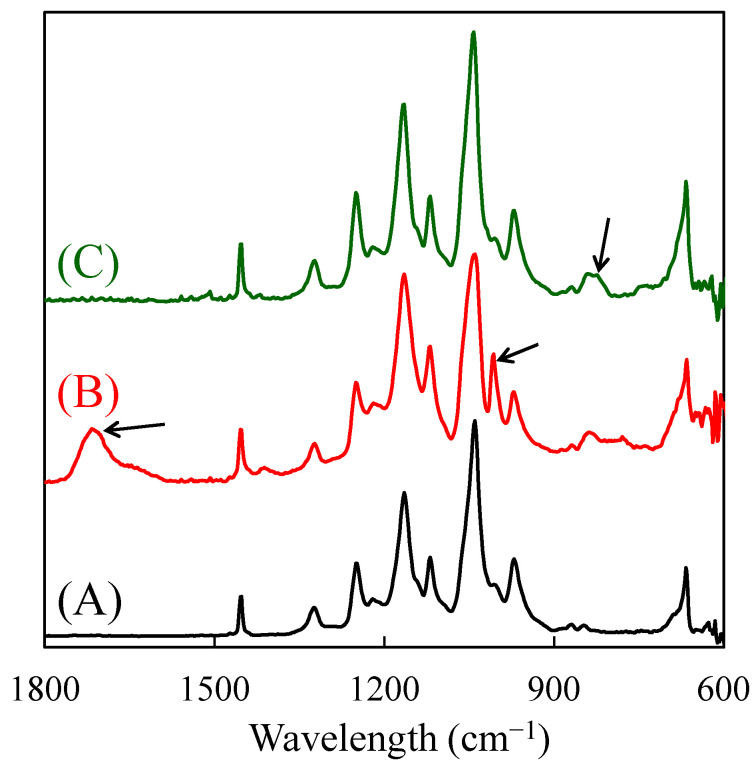
FT-IR spectra of the base ETFE film (A), SSS + acrylic acid (AA)-grafted side (B), and CMS-grafted side (C) of the grafted film prepared in Run 4 in Table 4.

Figure 5 shows the FT-IR spectra of the base ETFE film and SSS + AA- and CMS-grafted sides of the film prepared in Run 4. In the SSS + AA-grafted side, the characteristic two peaks were observed at 1007 and 1707 cm^−1^. The former was attributed to SO_2_ symmetric stretching from SO_3_Na of SSS units [27], and the latter was attributed to C=O stretching from COOH of AA units [28]. In the CMS-grafted side, the abovementioned two peaks were not observed; instead, the peak attributed to C–Cl stretching from CMS units was observed at 820 cm^−1^ [29]. These FT-IR spectra also support the clear separation of SSS + AA- and CMS-grafted layers. 

#### 3.2.2. RIAGP with Time Lag

Figure 4D shows that the thickness of the SSS + AA-grafted layer (20 μm) is thin compared to that of the CMS-grafted layer (56 μm). Thus, the obtained BPM will consist of a narrow CEL and wide AEL. This occurs owing to the difference in grafting speeds: the penetration of the hydrophilic SSS + AA solution into the hydrophobic base ETFE film was slower than that of the hydrophobic CMS solution. During the BPM fuel cell operation, H^+^ formed at the anode moves through the CEL, and OH^−^ formed at the cathode moves through AEL. Then, H^+^ and OH^−^ unite to form H_2_O at the CEL/AEL boundary. If the AEL is considerably thicker than the CEL, the OH^−^ transport in the AEL requires more time and becomes the dominant rate-determining step that restricts the output power. 

Accordingly, we tried to control the balance of the thicknesses of the SSS + AA- and CMS-grafted layers by the time-lag RIAGP method, as shown in Run 5 in Table 4. Similar to Run 4, the ETFE film irradiated with 80-kGy γ-rays was placed in the reaction cell, and then an SSS + AA/water solution was injected into the left compartment of the cell. The right compartment was kept empty, and the reaction cell was placed in an oven at 60 °C to initiate the grafting polymerization of SSS + AA. After 3.5 h, a CMS/xylene solution was injected into the right compartment, and the cell was kept at 60 °C for additional 6 h. The obtained grafted film had a thickness of 64 μm and *DOG_eff_* of 85.6%. 

#### 3.2.3. Amounts of Sulfonic Acid and Carboxyl Groups in BPM

Figure 4D shows that a clear boundary between two grafted layers can be formed by selecting the monomer/solvent combinations of SSS + AA/water and CMS/xylene, which corresponds to Route (iii) in Figure 2 for BPM preparation. This BPM has two ionic groups, i.e., sulfonic acid (SO_3_H) and carboxylic acid (COOH). Their amounts, X_SO3H_ and X_COOH_, were determined as follows. The film was cut into a 3 × 4 cm^2^ piece and immersed in a 1-mol/L HCl aqueous solution at 25 °C for 24 h. Then, the film was immersed in a 3-mol/L NaCl aqueous solution at 25 °C for 24 h to change the counter ions of SO_3_H from H^+^ to Na^+^. In this solution at pH 7.0, the counter ion of COOH did not change and remained H^+^. The amount of H^+^ liberated into the solution was titrated with NaOH to determine X_SO3__H_. Then, the film was immersed in a 1-mol/L HCl aqueous solution at 25 °C for 24 h and stored in deionized water. 

Next, the grafted film was immersed in 15 mL of a 0.1-mol/L NaOH aqueous solution at 25 °C for 24 h. Under this alkaline condition, both acids (SO_3_H and COOH) were converted from H^+^ to Na^+^ forms by consuming NaOH in the solution. The amount of consumed NaOH, X_NaOH_, was evaluated as the difference between the initial and remaining amounts of NaOH that was titrated with HCl. The value of X_COOH_ was calculated by subtracting X_SO3H_ from X_NaOH_. 

### 3.3. Electrolyte Properties of Prepared BPMs 

**Figure 6:**
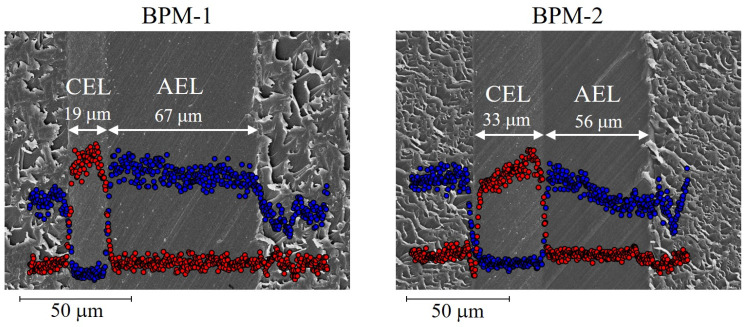
SEM images and concentration profile of sulfur (red circle) and chlorine (blue circle) of the cross-section of the prepared BPMs.

ETFE-based SSS + AA/CMS-grafted films were immersed in a TMA solution to introduce quaternary ammonium groups [N(CH_3_)_3_^+^]. BPMs prepared in Runs 4 and 5 in Table 4 were referred to as BPM-1 and BPM-2, respectively. Figure 6 shows the SEM images of the cross-section of BPMs and the concentration profile of sulfur and chlorine (counter ion of N(CH_3_)_3_^+^) in the CEL and AEL. For BPM-1, similar to its precursor grafted film (see Figure 4D), sulfur was observed only in the left thin layer, and chlorine was observed only in the right wide layer. Accordingly, the left and right layers are assumed to be the CEL and AEL, respectively. This was the first example of the creation of a BPM with a clear CEL/AEL boundary prepared by RIAGP. The thickness of the AEL (67 μm) was larger than that of the CMS-grafted layer (56 μm) shown in Figure 4D because its volume was enlarged by quaternarization. 

For BPM-2, a clear CEL/AEL boundary was also observed. Compared to BPM-1, the CEL thickness increased from 19 to 33 μm by time-lag RIAGP, in which SSS + AA graft polymerization was performed for a longer time. It was demonstrated that the thicknesses of the CEL and AEL can be controlled using an appropriate time lag. 

The amount of N(CH_3_)_3_^+^ group in BPMs, X_N+(CH3)3_, was determined as follows. A BPM was immersed in a 1 mol/L KOH aqueous solution so that the counter ions of cation exchange groups (SO_3_H and COOH) and anion exchange groups [N^+^(CH_3_)_3_] changed to K^+^ and OH^−^, respectively. The BPM was immersed in 15 mL of a 0.1 mol/L HCl aqueous solution at 25 °C for 24 h, and the counter ions of cation and anion exchange groups changed to H^+^ and Cl^−^, respectively. This ion exchange reaction accompanied the HCl consumption in a solution. The amount of consumed HCl, X_HCl_, was the difference in the initial and remaining amounts of HCl that was titrated with NaOH. The value of $X_{{N{\left(CH_{3}\right)}_{3}}^{+}}$ was calculated by subtracting X_SO3H_ and X_COOH_ from X_HCl_. This H^+^/Cl^−^ form BPM was immersed in deionized water and vacuum-dried. *CEC* and *AEC* were calculated using:

(4) $$CEC\;\left(\mathrm{mmol}/g\right)\;=\;X_{SO_{3}H}/W_{D}$$

(5) $$AEC\;\left(\mathrm{mmol}/g\right)\;=\;X_{{N{\left(CH_{3}\right)}_{3}}^{+}}/W_{D}$$

where *W_D_* is the dried BPM weight, as defined in Equation (2). 

Table 5 shows the properties of BPMs. The *CEC* and *AEC* of BPM-1 were 0.35 and 1.48 mmol/g, respectively. The lower *CEC* compared to *AEC* was in good agreement with a smaller thickness of the CEL. The *CEC* of BPM-2 was twice as high (0.67 mmol/g) owing to a wider CEL. The SSS ratio in SSS + AA grafts, *R_SSS_*, was calculated using:

(6) $$R_{SSS}=\;100\;X_{SO_{3}H}/\;\left(X_{SO_{3}H}+\;X_{COOH}\right)$$

The *R_SSS_* values of BPM-1 and BPM-2 were 39.6 and 52.5%, respectively. The higher the *R_SSS_* is, the higher is the efficiency of cation transport because AA units do not contribute to cation conduction in the CEL. A proper condition for introducing a larger amount of SSS compared to AA in their co-grafting polymerization is being examined. 

In a future work, we will apply novel BPMs prepared by RIAGP to fuel cell power generation experiments. The clear CEL/AEL boundary is expected to efficiently form water (H^+^ + OH^−^ → H_2_O), which would result in high output power even under dry conditions. One concern is that the prepared BPM may not have enough long-term chemical stability during the fuel cell operation. This is because the AEL contains quaternary ammonium groups, which are gradually degraded by the Hofmann elimination and/or nucleophilic substitution in typical AEMs [10]. In contrast, these degradations can be restricted in AEMs containing the graft chains with various types of imidazolium groups as anion exchange groups [30,31]. Thus, we will consider forming the AEL with imidazolium groups by RIAGP to prepare BPMs with high chemical stability. 

## 4. Conclusions

Novel BPMs with a clear CEL/AEL boundary were prepared by RIAGP, in which different monomers were grafted at the same time into the base film from both sides. The grafting reaction behavior inside the film was carefully investigated by choosing various monomer/solvent combinations. For the combinations of SSS/DMSO and CMS/xylene or EtSS/DMSO and CMS/xylene, two grafted regions were mixed and were not clearly separated. By contrast, when the combinations of SSS + AA/water and CMS/xylene were chosen for grafting into ETFE, SSS + AA- and CMS-grafted regions were clearly separated. This occurred owing to the immiscibility between hydrophilic SSS + AA and hydrophobic CMS solutions. By the quaternization of CMS units, a BPM with a clear CEL/AEL boundary was prepared for the first time. In this BPM, the CEL was thinner than the AEL owing to the slow speed of grafting of hydrophilic SSS + AA monomers into the hydrophobic ETFE film. Then, time-lag RIAGP was performed, in which SSS + AA was first grafted from one side of the base film and, after a planned time, CMS was grafted from the opposite side. The CEL in the BPM was thicker than that in the BPM prepared with no time lag. Accordingly, the thicknesses of both layers can be controlled using time-lag RIAGP. In a future work, we will apply novel BPMs with a clear CEL/AEL boundary to fuel cells and will evaluate their performance under dry conditions. 

## Figures and Tables

**Table 1:** Log (octanol/water partition coefficient (P_OW_)) of several solvents [26].

Solvents	Log (P_OW_)	Solvents	Log (P_OW_)
dimethyl sulfoxide (DMSO)	−1.35	ethanol	−0.31
water	−1.15	1-propanol	0.25
*N*-methylpyrrolidone (NMP)	−0.54	toluene	2.73
methanol	−0.50	xylene	3.15

**Table 2:** Dissolution behavior of four monomers in various solvents.

Monomer and Its Concentration	Solvent	Solubility
SSS 0.8 mol/L	water	○
SSS 0.8 mol/L	DMSO	○
SSS 1.0 mol/L	water	×
SSS 1.0 mol/L	DMSO	○
SSS 1.0 mol/L + AA 0.6 mol/L	water	×
SSS 1.0 mol/L + AA 0.8 mol/L	water	○
SSS 1.2 mol/L + AA 0.8 mol/L	water	×
EtSS 50 vol%	water	×
EtSS 50 vol%	DMSO, xylene	○
CMS 50 vol%	water	×
CMS 50 vol%	DMSO, xylene	○

Symbols ○ and × mean complete and incomplete dissolution, respectively.

**Table 3:** Results of the radiation grafting of monomers into various base films.

**Base Film**	Monomer and Its Concentration	Solvent	*DOG* (%)
PVDF	SSS 0.8 mol/L	water	0
	SSS 1.0 mol/L	DMSO	46.7
	SSS 1.0 mol/L + AA 0.8 mol/L	water	165
	EtSS 50 vol%	DMSO	212
	EtSS 50 vol%	xylene	4.19
	CMS 50 vol%	DMSO	67.6
	CMS 50 vol%	xylene	54.1
ETFE	SSS 0.8 mol/L	water	0
	SSS 1.0 mol/L	DMSO	0.59
	SSS 1.0 mol/L + AA 0.8 mol/L	water	240
	EtSS 50 vol%	DMSO	38.0
	EtSS 50 vol%	xylene	20.3
	CMS 50 vol%	DMSO	105
	CMS 50 vol%	xylene	114
PE	SSS 0.8 mol/L	water	0
	SSS 1.0 mol/L	DMSO	0.55
	SSS 1.0 mol/L + AA 0.8 mol/L	water	131.4
	EtSS 50 vol%	DMSO	208
	EtSS 50 vol%	xylene	290
	CMS 50 vol%	DMSO	49.3
	CMS 50 vol%	xylene	67.1
Nylon 6	SSS 0.8 mol/L	water	251
	EtSS 50 vol%	DMSO	33.6
	EtSS 50 vol%	xylene	0
	CMS 50 vol%	DMSO	0
	CMS 50 vol%	xylene	0.73

**Table 4:** Experimental conditions of RIAGP and obtained effective degree of grafting (*DOG_eff_*)_._

Run	Base Film	Monomer 1 and Its Concentration	Solvent of Monomer 1	Monomer 2 and Its Concentration	Solvent of Monomer 2	Time-Lag (h)	Thickness —μm)	*DOG_eff_* (%)
1	PVDF	SSS 1.0 mol/L	DMSO	CMS 50 vol%	xylene	0	56	26.4
2	PVDF	EtSS 50 vol%	DMSO	CMS 50 vol%	xylene	0	71	121
3	ETFE	EtSS 50 vol%	DMSO	CMS 50 vol%	xylene	0	72	95.8
4	ETFE	SSS 1.0 mol/L + AA 0.8 mol/L	water	CMS 50 vol%	xylene	0	73	125
5	ETFE	SSS 1.0 mol/L + AA 0.8 mol/L	water	CMS 50 vol%	xylene	3.5	64	85.6

**Table 5:** Properties of the prepared BPMs.

	BPM-1	BPM-2
RIAGP condition listed in Table 4	Run 4	Run 5
Membrane thickness (μμm)	86	89
Thickness of the CEL (μm)	19	33
Thickness of the AEL (μm)	67	56
*CEC* (mmol/g)	0.35	0.67
*AEC* (mmol/g)	1.48	1.19
*R_SSS_* (%)	39.6	52.5
Water uptake (%)	76.1	73.5

## Data Availability

The data described in the manuscript are available from the corresponding author on reasonable request.

## Sample Availability

Samples of the compounds are not available from the authors.
